# Effect of Th17 and Treg Axis Disorder on Outcomes of Pulmonary Arterial Hypertension in Connective Tissue Diseases

**DOI:** 10.1155/2014/247372

**Published:** 2014-08-20

**Authors:** Saren Gaowa, Wenyong Zhou, Lilei Yu, Xiaohui Zhou, Kai Liao, Kang Yang, Zhibin Lu, Hong Jiang, Xiaofeng Chen

**Affiliations:** ^1^Department of Cardiology, Renmin Hospital of Wuhan University, Wuhan, Hubei 430060, China; ^2^Department of Cardiothoracic Surgery, East Hospital, Tongji University, Shanghai 200120, China; ^3^Department of Cardiothoracic Surgery, Huashan Hospital, Fudan University, Shanghai 200040, China

## Abstract

This prospective cohort study is to verify the hypothesis that the balance of Th17 and Treg cells frequencies in the peripheral circulation is disturbed in patients with varying degrees of connective tissue diseases-associated pulmonary arterial hypertension (CTD-aPAH) and to prove the influence of Th17/Treg imbalance on prognosis. We detected the frequencies and absolute counts of Th17 and Treg cells and related serum cytokines secretion and expressions of key transcription factors in 117 patients with connective tissue diseases (CTD), 53 patients with CTD-aPAH, and 48 healthy volunteers. Moreover, the median value according to levels of Th17/Treg ratios in patients with CTD-aPAH was chosen as basis of group division for survival analysis. CTD-aPAH patients revealed significant increase in peripheral Th17 cells, Th17-related cytokines, and ROR *γ*t mRNA levels. They also presented a significant decrease in Treg cells, Treg-related cytokines, and Foxp3 mRNA levels as compared with CTD patients and healthy controls. More importantly, the Th17/Treg ratio was significantly related to the severity and prognosis of CTD-aPAH. This study indicated that the Th17/Treg axis disorder plays a critical role in CTD-aPAH. Furthermore, the dynamic balance between Th17 and Treg cells was likely to influence prognosis of patients with CTD-aPAH.

## 1. Introduction

Pulmonary arterial hypertension (PAH) is a common complication of connective tissue diseases (CTD) such as systemic sclerosis (SSc), systemic lupus erythematosus (SLE), mixed connective tissue disease (MCTD), rheumatoid arthritis (RA), and primary Sjogren's syndrome (pSS). In updated clinical classification of pulmonary hypertension [1], this type of pulmonary hypertension (PH) was defined as follows: PAH associated with connective tissue diseases belongs to Group One. Most of CTD-associated PAH (CTD-aPAH) presented itself as the type of SSc-associated PAH (SSc-aPAH). The prevalence of PAH in patients with SSc, on the condition that the diagnosis is based on right heart catheterization for assessment of filling pressures, is about 8–14% [2, 3]. Not only the prevalence of CTD-aPAH is higher than idiopathic PAH (IPAH), but also its prognosis is worse than IPAH [4, 5].

Th17 cells expressing retinoic acid-related orphan receptor *γ*t (ROR *γ*t) are a recently-identified subset of CD4^+^ T cells. Meanwhile, this cell subset, having the capacity to produce potent proinflammatory cytokines including IL-17a (IL-17), IL-17F, IL-21, and IL-22, is likely to play an important role in autoimmunity [6, 7], while CD4^+^CD25^+^Foxp3^+^regulatory T cells (Treg) cells expressing forkhead/winged helix transcription factor (Foxp3) are capable of modulating the function of effector T cells, maintaining immunological homeostasis, and preventing autoimmunity [8, 9]. However, the detection of Tregs by specific surface marker staining (CD4^+^CD25^+^ CD127^−^) is also widely-used in recent researches [10, 11]. A fine balance between effector T cells and Treg cells regulates immune homeostasis. The dynamic balance between Th17 and Treg cells involved in the pathogenesis of CTD is damaged [12–15].

It is acknowledged that a possible role of the Th17/Treg axis in CTD-aPAH has never been elucidated. Besides, the relevant researches about the prognosis influence of Th17/Treg axis disorder as pathogenic factor on patients with CTD-aPAH remain to be discovered. We hypothesized that imbalance of circulating Th17/Treg cells may also present themselves in patients with CTD-aPAH, which is related with pathogenesis of CTD-aPAH and important to the prognosis. Thus, the purpose of this prospective cohort study is to verify the hypothesis that the balance of Th17 and Treg cells frequencies and absolute counts in the peripheral circulation is disturbed in patients with varying degrees of CTD-aPAH and to prove the influence of Th17/Treg imbalance on prognosis. The plasma levels of Th17- and Treg-related cytokines and mRNA expression of relevant transcription factors (ROR *γ*t and Foxp3) in peripheral blood mononuclear cells (PBMCs) were evaluated and the potential correlation of Th17/Treg cells imbalance with the severity and prognosis of CTD-aPAH was also explored. N-terminal probrain natriuretic peptide (NT-proBNP), as a sensitive marker of cardiovascular diseases, is independently associated with mortality in IPAH and SSc-aPAH [16, 17]. Therefore, the potential correlation of Th17/Treg imbalance with serum NT-proBNP level of CTD-aPAH was explored.

## 2. Materials and Methods

### 2.1. Patients Selection

307 patients with CTD were prospectively studied from January 2010 to October 2012. CTD was classified into subtypes such as SSc, SLE, MCTD, RA, and pSS according to the American College of Rheumatology criteria. All patients were evaluated in the aspects of renal, cardiac, hepatic, and pulmonary functions. Chest high-resolution computed tomography (HRCT) was used to detect the existence of pulmonary embolisms or pulmonary interstitial disease. Patients who meet the following criteria were excluded from this research. Being younger than 18 years or older than 75 years; with CTD being at active stage; with a history of chronic infection or recent (<1 month) infection with clinically significant or inflammatory conditions including trauma, vaccination, any invasive medical tests or surgery (<3 months); with heart function belonging to World Health Organization functional class IV; with other comorbidities, such as severe hepatic or kidney failure, pulmonary fibrosis, obstructive sleep apnoea syndrome, cancer, uncontrolled hypertension, diabetes, obesity (body mass index (BMI) > 30 kg/m^2^), asthma, chronic obstructive pulmonary disease, inflammatory bowel disease, and pregnancy; being treated with corticosteroid or other immunosuppressant in last three months. The rest of the potential subjects who haven't met the exclusion criteria were checked with hemodynamics. Systolic pulmonary artery pressure (sPAP) > 40 mmHg, estimated by echocardiograph, was used as a screening threshold for catheterization. Patients identified by noninvasive tests were operated with catheterization of right side of the heart. Confirmed PAH was defined as mean pulmonary artery pressure (mPAP) ≥ 25 mmHg, with a pulmonary capillary wedge pressure of ≤15 mmHg at a resting state by using a catheter method. Fifty-three patients with PAH, whose leading causes were excluded such as inborn diseases, medicines, and infection, examined by catheter check, were admitted into CTD-aPAH group. Subjects with CTD-aPAH were classified into two subgroups according to their systolic pulmonary artery pressure as mild to moderate CTD-aPAH (40 < sPAP ≤ 70 mmHg, *n* = 22) and severe CTD-aPAH (sPAP > 70 mmHg, *n* = 31). The remaining 117 patients with CTD, whose pulmonary artery pressures were at normal state, were admitted into CTD group. 6-minute walk test and World Health Organization functional class statistics from two groups were recorded. The main characteristics of patients were summarized in Table 1. Forty-eight healthy volunteers with the feature of matched sex and age (39 females, 9 males, 61.9 ± 10.6 years old) were recruited into control group. This study was approved by the Institutional Review Board of Fudan University. All patients gave their written-informed consents.

### 2.2. Blood Sample Preparation

Peripheral blood mononuclear cells (PBMCs) were isolated from heparinized peripheral venous blood by Ficoll-Hypaque lymphoprep (Nyegaard, Norway) density centrifugation for analysis of flow cytometry and real-time quantitative polymerase chain reaction (RT-qPCR). Plasma was obtained after centrifugation and stored at −80°C for assay of the cytokines and NT-proBNP.

### 2.3. Flow Cytometric Analysis of Treg and Th17 Cells

For analysis of Treg cells, PBMCs were surface-labeled with CD4-PE/Cy5, CD25-PE followed by fixation and permeabilization and intracellularly stained with Foxp3-Alexa Fluro488 or were surface-labeled with CD4-PE/Cy5, CD25-PE, and CD127-FITC (eBioscience, USA).

For analysis of Th17 cells, PBMCs was suspended in complete culture medium (RPMI1640 was supplemented with 10% heat-inactivated fetal calf serum, (Gibco BRL, USA)). Cultures were stimulated for 1 hour using 50 ng/mL phorbol myristate acetate (PMA) and 1 *μ*g/mL ionomycin (Sigma-Aldrich, USA) and then stimulated using 500 ng/mL monensin (Alexis Biochemicals, USA) for 3 hours at environment of 37°C and 5% CO_2_. Cells were then washed in PBS and surface-labeled with CD4-PE/Cy5 (eBioscience, USA). Following surface staining, cells were fixed and permeabilized using fixation/permeabilization buffer (eBioscience, USA) and then stained with IL-17a-PE (eBioscience, USA). Labeled cells were washed and analyzed with FACS Calibur flow cytometer (Becton-Dickinson, USA) using FlowJo (Tree Star, USA) v7.6.1 software. In each case, staining was compared with that of the appropriately labeled isotype control antibody.

### 2.4. Transcription Factors Expression Determined by Real-Time Quantitative PCR

Total RNA was extracted from individual PBMCs preparation by using TRIzol reagent (Invitrogen, USA). cDNA was prepared from total RNA extraction by reverse transcription with oligo(dT). The following primer pairs were used: Foxp3: F: 5′-CACGCATGTTTGCCTTCTTCAGA-3′; R: 5′-GTAGGGTTGGAACACCT GCTGGG-3′; and ROR *γ*t: F: 5′-GCAATGGAAGTGGTGCTGGTT-3′; R: 5′-AGGATGCTTTGGCGATGAGTC-3′. Real-time quantitative PCR for Foxp3, ROR *γ*t were performed in ABI Prism 7900 Sequence Detection System (Applied Biosystems, USA) with the SYBR green master mix kit (Takara, China). Both Foxp3 and ROR *γ*t expression data were, then, normalized relative to glyceraldehyde-3-phosphate dehydrogenase (GAPDH).

### 2.5. Detection of Plasma Cytokines and NT-proBNP

The plasma levels of IL-17a, IL-6, IFN-*γ*, IL-2, TGF-*β*, and TNF-*β* were measured by radioimmunoassay method utilizing radioimmunoassay kit (NIBT, China), abiding by the protocol of department of nuclear medicine of Huashan hospital, Fudan University. All samples were measured in duplicates.

As previously described [17], the NT-proBNP concentration was determined with the method of an Elecsys NT-proBNP sandwich immunoassay by an Elecsys 2010 instrument (Roche Diagnostics, Switzerland). The analytical range was extended from 20 pg/mL to 35000 pg/mL.

### 2.6. Statistical Analysis

Continuous variables were expressed as mean ± standard deviations. Dichotomous variables were expressed as percentages. Comparisons among groups were made using Students'* t*-test or Pearson chi-square test. Differences among the multiple groups were analyzed with ANOVA followed by the Scheffe test. Spearman's rank sum test was used to analyze correlation coefficients. Nonparameter analysis was performed by using Kolmogorov-Smirnov (K-S) test to analyze the distribution. Survival analysis was prepared by using the Kaplan-Meier method. The log-rank test was used to compare the difference. *P* value < 0.05 was considered as significant difference. Statistics were analyzed by using the SPSS17.0 Statistics SoftwarePackage (SPSS Inc., USA).

## 3. Results

### 3.1. Percentages of Treg, Th17 Cells in the Peripheral Blood of Patients with CTD or CTD-aPAH

**Figure 1:**
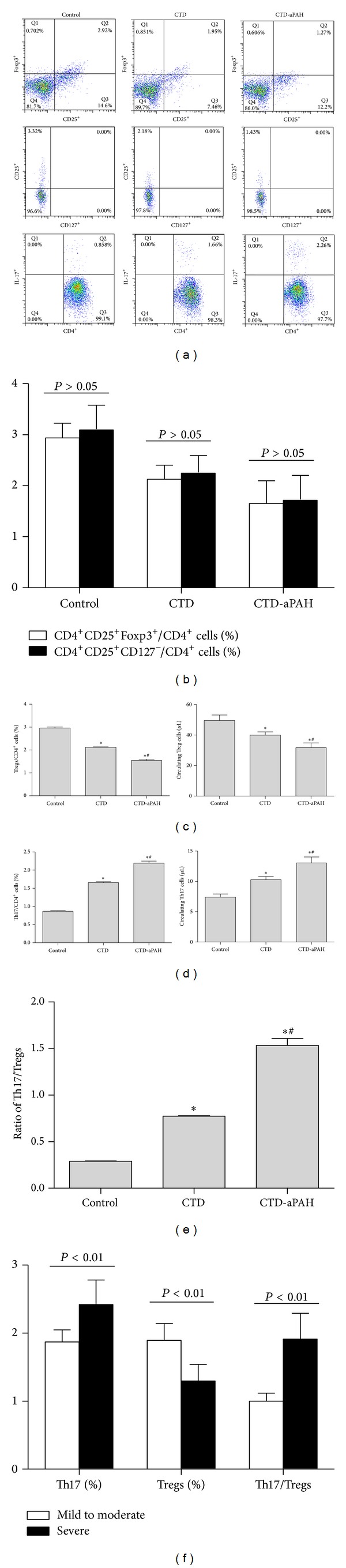
Frequencies and absolute counts of circulating Treg and Th17 cells as well as the ratio of Th17/Treg in CTD and CTD-aPAH patients and healthy controls. PBMCs from studied subjects were stained with labeled anti-human antibodies as described in Section 2. Flow cytometry dot-plots showed that the gating strategies were used in identification of Treg (gated on CD4^+^ cells for detection of CD4^+^ CD25^+^ Foxp3^+^ and CD4^+^CD25^+^ CD127^−^ cells) and Th17 cells (gated on CD4^+^ cells for detection of CD4^+^IL-17^+^ cells). (a) The dot-plots showed the gating strategies of circulating CD4^+^CD25^+^ Foxp3^+^ Treg, CD4^+^CD25^+^ CD127^−^ Treg and Th17 cells in control, CTD and CTD-aPAH groups. (b) Statistical analysis revealed the frequencies of CD4^+^ CD25^+^ Foxp3^+^ Treg and CD4^+^ CD25^+^ CD127^−^ Treg populations among the CD4^+^ T cells in healthy controls (*n* = 20) and in patients with CTD (*n* = 18) or CTD-aPAH (*n* = 23). (c) Frequencies (left) and absolute counts (right) of circulating Treg cells (CD4^+^CD25^+^ Foxp3^+^) in control, CTD and CTD-aPAH groups. (d) Frequencies (left) and absolute counts (right) of circulating Th17 cells in control, CTD and CTD-aPAH groups. (e) The ratios of Th17/Treg in control, CTD and CTD-aPAH groups. (f) Th17 and Treg cells frequencies and the ratios of Th17/Treg in subgroups of mild to moderate CTD-aPAH and severe CTD-aPAH. *Compared with control group, *P* < 0.05; ^#^Compared with CTD group, *P* < 0.05.

We compared the discrimination of Tregs by detection of CD4^+^CD25^+^Foxp3^+^ T cells and CD4^+^CD25^+^CD127^−^ T cells but could not detect significant differences between both methods which were utilized in 18 patients with CTD, 23 patients with CTD-aPAH, and 20 healthy controls (*P* > 0.05) (as shown in Figures 1(a) and 1(b)). We utilized CD4, CD25 and Foxp3 as the markers to detect Treg cells in this study. The prevalence of Treg cells was expressed as a ratio of CD4^+^CD25^+^Foxp3^+^/CD4^+^ T cells and absolute counts.

As shown in Figure 1(c), the frequencies and absolute counts of Treg cells were significantly decreased in the peripheral blood of patients with CTD (2.12 ± 0.20%; 39.97 ± 22.98 cells/*μ*L) and CTD-aPAH (1.55 ± 0.38%; 28.30 ± 21.01 cells/*μ*L) compared with those of healthy controls (2.96 ± 0.29%,*P* < 0.01, *P* < 0.01; 53.40 ± 25.35 cells/*μ*L, *P* < 0.01, *P* < 0.01). Circulating Treg cells percentages and absolute counts were markedly higher in patients with CTD than those patients with CTD-aPAH (2.12 ± 0.20% versus 1.55 ± 0.38%, *P* < 0.01; 39.97 ± 22.98 cells/*μ*L versus 28.30 ± 21.01 cells/*μ*L, *P* = 0.011). Moreover, significant differences of percentages were found between patients with severe CTD-aPAH and patients with mild to moderate CTD-aPAH (1.30 ± 0.24% versus 1.90 ± 0.25%, *P* < 0.01) (as shown in Figure 1(f)).

As shown in Figures 1(a) and 1(d), the prevalence of Th17 cells was expressed as a ratio of CD4^+^ IL-17^+^T cells/CD4^+^ T cells and absolute counts. The frequencies and absolute counts of Th17 cells were evidently increased in the peripheral blood of patients with CTD (1.65 ± 0.28%; 10.29 ± 5.52 cells/*μ*L) and CTD-aPAH (2.19 ± 0.40%; 13.06 ± 7.19 cells/*μ*L) than those in normal control group (0.87 ± 0.14%, *P* < 0.01, *P* < 0.01; 7.40 ± 3.60 cells/*μ*L, *P* = 0.012, *P* < 0.01). Significant differences of percentages and absolute counts were also found between CTD-aPAH and CTD group (2.19 ± 0.40% versus 1.65 ± 0.28%, *P* < 0.01; 13.06 ± 7.19 cells/*μ*L versus 10.29 ± 5.52 cells/*μ*L, *P* = 0.013). Furthermore, the percentages of Th17 cells were markedly higher in patients with severe CTD-aPAH than those in subgroup with mild to moderate CTD-aPAH (2.42 ± 0.36% versus 1.87 ± 0.18%, *P* < 0.01) (as shown in Figure 1(f)).

### 3.2. Imbalance of Circulating Th17/Treg Cells in Patients with CTD or CTD-aPAH

The significance of increased Th17 cells and decreased Treg cells was further explored by calculation of Th17/Treg cells percentage ratio of patients in CTD-aPAH, CTD, and healthy controls groups (as shown in Figure 1(e)). We demonstrated that the ratio of Th17 to Treg cells was highest in patients with CTD-aPAH (1.53 ± 0.54), lower in those patients with CTD (0.77 ± 0.06) and lowest in subjects of control group (0.29 ± 0.02). As a result, the Th17/Treg cells ratio was significantly increased in patients with CTD-aPAH compared with patients in CTD and control group (1.53 ± 0.54 versus 0.77 ± 0.06, *P* < 0.01; 1.53 ± 0.54 versus 0.29 ± 0.02, *P* < 0.01). In CTD-aPAH group, the similar significant difference also presented in two subgroups between severe CTD-aPAH and mild to moderate CTD-aPAH (1.91 ± 0.38 versus 1.00 ± 0.12, *P* < 0.01) (as shown in Figure 1(f)).

### 3.3. Expression of ROR *γ*t and Foxp3 mRNA in PBMCs of Patients with CTD or CTD-aPAH

**Figure 2:**
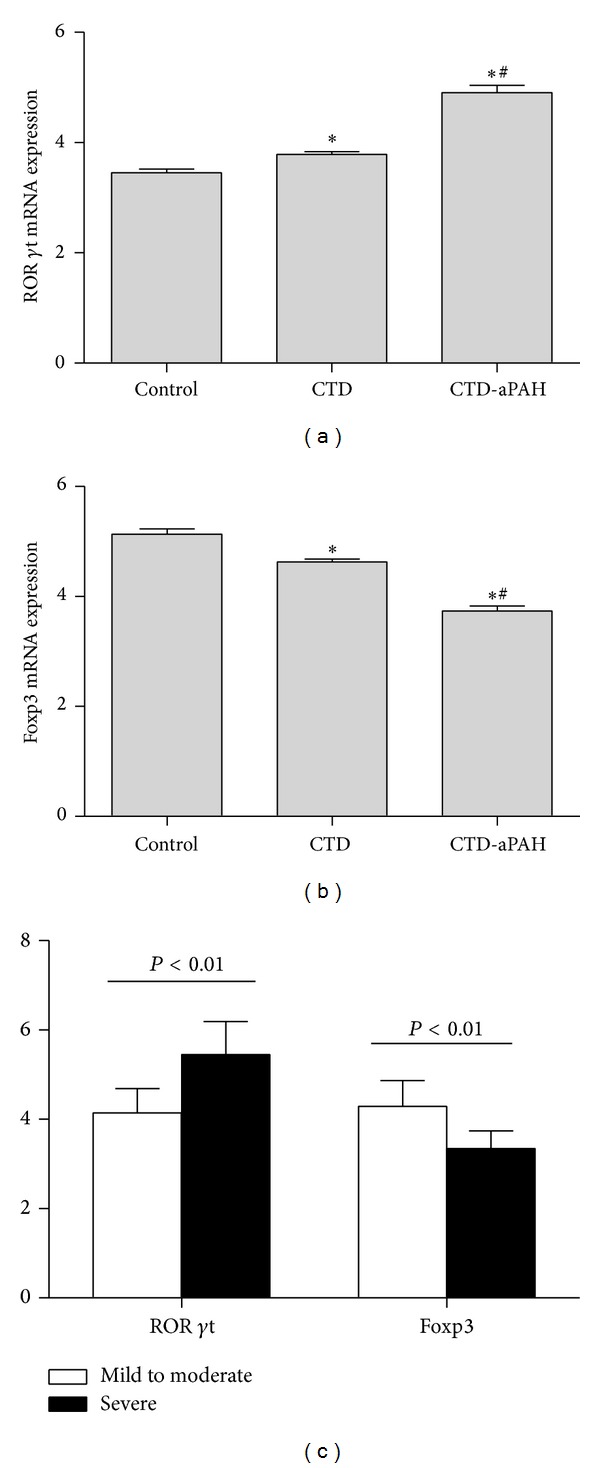
Expression of ROR *γ*t and Foxp3 mRNA in CTD and CTD-aPAH patients and healthy controls. mRNA expression in PBMCs from studied subjects was measured by real-time quantitative polymerase chain reaction. The result was normalized relative to GAPDH. (a) The expression of ROR *γ*t mRNA in CTD, CTD-aPAH, and control group. (b) The expression of Foxp3 mRNA in CTD, CTD-aPAH, and control group. (c) The expression of ROR***γ***t and Foxp3 mRNA in subgroups of mild to moderate CTD-aPAH and severe CTD-aPAH. *Compared with control group, *P* < 0.01; ^#^Compared with CTD group, *P* < 0.01.

The specific transcription factors of both T subsets in CTD-aPAH, CTD, and control group were evaluated to confirm cytology observations. ROR *γ*t is an important transcription factor for the differentiation of Th17 cells, while Foxp3 is the master transcription factor in Treg cells. The levels of these transcription factors for Th17 and Treg cells were analyzed in PBMCs from included subjects by RT-qPCR. As shown in Figure 2(a), the levels of ROR *γ*t expression were upregulated in CTD group (3.79 ± 0.52) and CTD-aPAH (4.91 ± 0.93) than those in the healthy control group (3.45 ± 0.45; *P* < 0.01, *P* < 0.01), while there was also significant difference between previous two groups (*P* < 0.01). In contrast, the expression of Foxp3 was markedly lower in patients with CTD (4.63 ± 0.61) and CTD-aPAH (3.74 ± 0.67) as compared with patients in the control group (5.13 ± 0.69; *P* < 0.01, *P* < 0.01). Furthermore, CTD-aPAH patients had a significantly lower expression than patients with CTD (*P* < 0.01) (as shown in Figure 2(b)). Moreover, ROR *γ*t and Foxp3 levels in mild to moderate CTD-aPAH subgroup (4.14 ± 0.55; 4.29 ± 0.58) were significantly different from those of the subgroup with severe CTD-aPAH (5.45 ± 0.74, *P* < 0.01; 3.34 ± 0.40, *P* < 0.01) (as shown in Figure 2(c)). These results were consistent with flow cytometric analysis of Th17 and Treg cells.

### 3.4. Th17- and Treg-Related Cytokines and NT-proBNP in the Serum from Patients with CTD or CTD-aPAH

T-helper and regulatory T cells-related cytokines and NT-proBNP in serum of patients in CTD and CTD-aPAH and healthy controls were measured by radioimmunoassay or Elecsys NT-proBNP sandwich immunoassay. As shown in Table 2, patients with CTD exhibited higher levels of serum IL-17a and NT-proBNP than those patients in healthy controls (*P* < 0.01) but lower than those patients with CTD-aPAH (*P* < 0.01). Meanwhile, the expression of TGF-*β* was highest in subjects of control group, lower in patients with CTD, and lowest in patients with CTD-aPAH. Patients in both CTD and CTD-aPAH group had higher levels of serum IL-6, IFN-*γ*, IL-2, and TNF-*α* when compared with patients in healthy controls (*P* < 0.01), while no significant difference was observed between CTD and CTD-aPAH group (*P* > 0.05). In subgroups, there were obvious differences between mild to moderate CTD-aPAH group and severe CTD-aPAH group on serum IL-17a, TGF-*β*, and NT-proBNP (*P* < 0.01).

### 3.5. Correlation of Th17/Treg Ratios with Related Cytokines, Expression of ROR *γ*t and Foxp3 mRNA, and Markers of Disease Severity in Patients with CTD-aPAH

**Figure 3:**
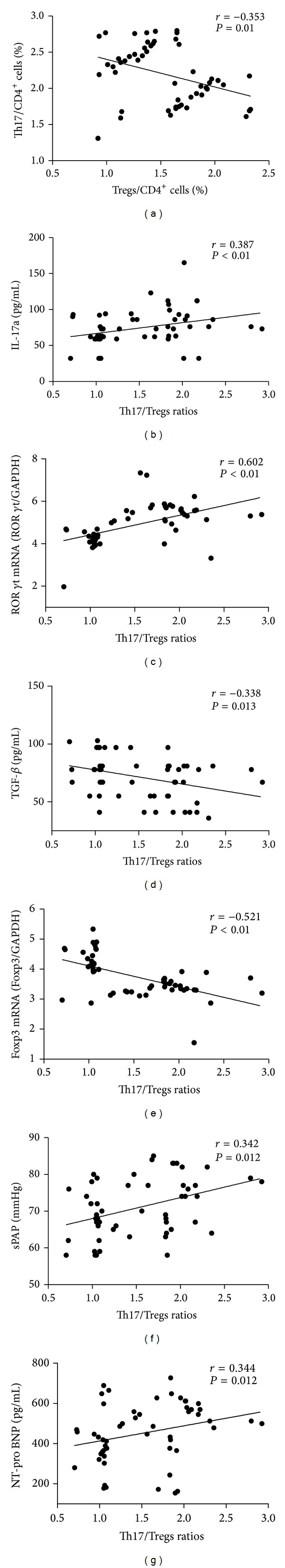
Correlation between Th17/Treg ratios and related cytokines; expression of ROR *γ*t and Foxp3 mRNA and markers of disease severity in patients with CTD-aPAH. (a) Ratios of Treg to CD4^+^ cells negatively correlate with ratios of Th17 to CD4^+^ cells. (b) Ratios of Th17/Treg positively correlate with IL-17 concentrations. (c) Ratios of Th17/Treg positively correlate with expression of ROR *γ*t mRNA. (d) Ratios of Th17/Treg negatively correlate with TGF-*β* concentrations. (e) Ratios of Th17/Treg negatively correlate with expression of Foxp3 mRNA. (f) Ratios of Th17/Treg positively correlate with levels of sPAP. (g) Ratios of Th17/Treg positively correlate with NT-pro BNP concentrations.

There were significant correlations between Th17 and Treg cells in the peripheral circulation of CTD-aPAH group (*r* = − 0.353, *P* = 0.01) (as shown in Figure 3(a)). Th17/Treg ratios were positively correlated with serum concentrations of IL-17a (*r* = 0.387, *P* < 0.01) and expression of ROR *γ*t mRNA (*r* = 0.602, *P* < 0.01) (as shown in Figures 3(b) and 3(c)) in PBMCs and negatively related with serum concentrations of TGF-*β* (*r* = − 0.338, *P* = 0.013) and expression of Foxp3 mRNA (*r* = − 0.521, *P* < 0.01) (as shown in Figures 3(d) and 3(e)) in PBMCs. The relationship between Th17/Treg ratios and serum levels of IL-6, IFN-*γ*, IL-2, and TNF-*α* in patients with CTD-aPAH was also studied, but no correlations were identified (*P* > 0.05).

sPAP and serum levels of NT-proBNP were used as markers of disease severity of patients with CTD-aPAH. In CTD-aPAH group, sPAP and serum levels of NT-proBNP were both positively correlated with ratio of Th17/Treg (*r* = 0.342, *P* = 0.012; *r* = 0.344, *P* = 0.012) (as shown in Figures 3(f) and 3(g)).

### 3.6. Survival Analysis

To evaluate Th17 and Treg axis association with clinical outcomes, Th17/Treg ratio was considered as a categorical variable. K-S test showed that Th17/Treg ratio in patients with CTD-aPAH exhibited skewed distribution (*P* = 0.032). Therefore, the median value (1.474), which was calculated as ratio of Th17/Treg of 53 patients in this research, was chosen as division standard for subgroup. Twenty-six patients with CTD-aPAH whose Th17/Treg ratios were less than 1.474 were admitted into low Th17/Treg ratio group. In contrast, 27 patients with CTD-aPAH whose Th17/Treg ratios were equal to or more than 1.474 were admitted into high Th17/Treg ratio group. The evaluation period was defined from the date that patients with CTD-aPAH were admitted into abovementioned two subgroups till October 2012. Patients who had survived overdue the observation period deadline were considered as censored.

**Figure 4:**
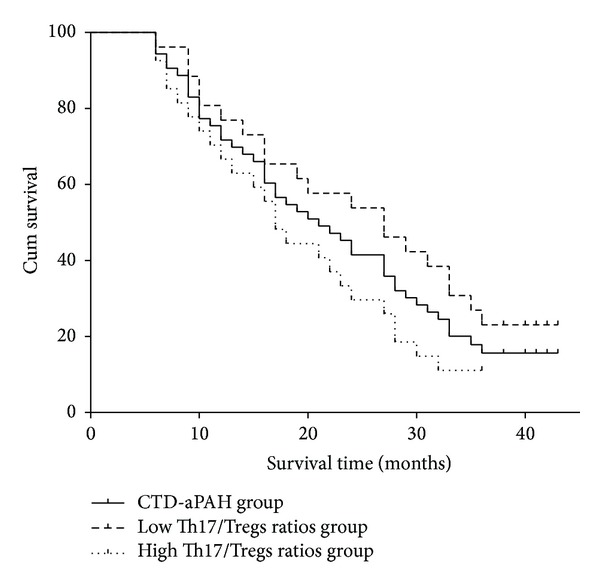
Kaplan-Meier survival analysis of patients with CTD-aPAH. Curves showed overall survival time for patients with CTD-aPAH who had lower or higher Th17/Treg ratios.

The overall survival time for all 53 patients with CTD-aPAH was 22.63 ± 1.64 months. For patients in low Th17/Treg ratios group, the overall survival time was 25.79 ± 2.46 months. For patients in high Th17/Treg ratios group, the overall survival time was 19.52 ± 2.00 months. For all patients, the overall survival rates were 71.70%, 41.51%, and 16.98% at 1, 2, and 3 years, respectively. For patients in low Th17/Treg ratios group, the overall survival rates were 76.92%, 53.85%, and 23.08% at 1, 2, and 3 years, respectively. For patients in high Th17/Treg ratios group, the overall survival rates were 66.67%, 29.63%, and 11.11% at 1, 2, and 3 years, respectively (as shown in Figure 4). There was a significant difference between the two subgroups (${\overset{-}{X}}^{2}=4.10$, *P* = 0.043).

## 4. Discussion

In the steady state, Th17 and Treg cells may remain in a dynamic balance. This balance is destroyed in autoimmune diseases such as CTD. Despite the similarities in hemodynamic perturbations, outcomes in patients with CTD-associated PAH differ significantly from other forms of PAH. This type of PAH contains inherent attributes which is on the basis of CTD. Previous studies have confirmed that IL-17 concentrations and Th17 cells were elevated in patients with CTD [15, 18, 19]. Most reports have shown that the prevalence of Treg weakened in PBMCs compartment in CTD, while some studies have reported normal or elevated Treg levels [20–23]. Nevertheless, it is generally thought that immune suppression capacity of Treg diminished in patients with CTD [24–29]. These discrepancies are likely to be related to the variability of the series of patients included in the different studies in relation to type and stage of disease as well as administered treatment.

Although the frequency of PAH in the patients with CTD was high, the researches about Th17/Treg axis in CTD-aPAH were very rare. Tamosiuniene et al. demonstrated that vascular endothelial growth factor receptor-2 (VEGFR-2) blockade induced significant pulmonary endothelial apoptosis in T cell-deficient rats but not in immune reconstituted (IR) rats. IR with CD4^+^ CD25^+^ T cell subset prior to vascular injury attenuated the development of PAH [30]. In addition, after exposing to endothelial cells, Treg cells upregulate the surface expression of programmed death-1 (PD-1) receptor as well as secretion of IL-10 and TGF-*β*, whose reactions promote vasodilatation [31], whereas more direct evidences showed that PH conditions are also associated with aberrant Treg cells numbers, activity or simply reduced CD4^+^ T cell numbers [30, 32]. In summary, these studies suggest that Treg cells function is normally to limit vascular injury and may play a protective role against the development of PAH. There is a lack of related researches in this field as for Th17 cells, which are closely related with inflammation or autoimmune disorder, as well as counter-interacting to Treg cells subset. In our research, Th17/Treg axis disorder in different stages of CTD-aPAH and efficiency of Th17/Treg imbalance as biomarker for predicting prognosis of patients with CTD-aPAH were explored.

The present statistics provide direct evidence that the frequencies and absolute counts of Treg cells and the expression of Foxp3 mRNA were significantly lower in patients with CTD-aPAH when compared to patients with CTD or healthy controls. Meanwhile, we found out that patients with CTD-aPAH exhibited a significant increase in peripheral Th17 numbers, Th17-related cytokines, and ROR *γ*t mRNA levels when compared to patients with CTD or healthy controls, even though in patients with CTD-aPAH, the frequencies of Th17/Treg cells, Th17-related cytokines, and ROR *γ*t/Foxp3 mRNA levels showed significant difference between mild to moderate CTD-aPAH subgroup and severe CTD-aPAH subgroup. In addition, the ratio of Th17/Treg cells had a linear positive correlation with CTD-aPAH severity indicated by sPAP and NT-proBNP. These results demonstrated that Th17/Treg dynamic balance involved in pathogenesis of CTD-aPAH. Therefore, the Th17/Treg ratio could be an important tool in detecting and describing various immunological conditions in patients with CTD-aPAH.

The cytokine milieu plays a pivotal role in the differentiation from naive CD4^+^T cells to Treg or Th17 cells [33–35]. In the present study, results showed that the concentrations of IL-6 and IL-17, both of which elevated inflammation, were all significantly higher in patients with CTD and CTD-aPAH, and IL-17 concentrations were positively correlated with Th17/Treg ratios.

Several large-scaled prospective studies have shown that sPAP and NT-proBNP levels are independent predictors of severity and prognosis of IPAH and CTD-aPAH. A highly-connected link between the ratios of Th17/Treg and sPAP or serum NT-pro BNP levels had been proved in this study. These evidences indirectly suggested that the ratio of Th17/Treg has been effective in evaluating the severity of CTD-aPAH and in predicting the prognosis of patients with CTD-aPAH. Epidemiological researches of Th17/Treg ratios in patients with CTD-aPAH were rare. The distribution frequency of Th17/Treg ratio through K-S test in patients with CTD-aPAH showed skewed distribution. We choose median values according to levels of Th17/Treg ratios in patients with CTD-aPAH as basis of group division in the survival analysis. Survival analysis showed that patients with CTD-aPAH, who have lower Th17/Treg ratios, have better survival tendency than the patients with higher Th17/Treg ratios. These findings directly suggested that the dynamic balance between Th17 and Treg cells was likely to influence prognosis of patients with CTD-aPAH and to evaluate prognosis of patients with CTD-aPAH as the predicting factor.

## 5. Conclusion 

Our study demonstrated that the imbalance of Th17/Treg existed in the peripheral blood of patients with CTD-aPAH. We also confirmed that the ratio of Th17/Treg not only correlated with inflammatory markers but also associated with severity and prognosis of CTD-aPAH. All these suggested a potential role for Th17/Treg imbalance in the pathogenesis and progression of CTD-aPAH.

The abovementioned results needed proving in larger cohorts. In addition, the further studies could be designed to observe the change of Th17/Treg axis in patients with CTD-aPAH at an active stage and to investigate the impact of corticosteroid or immune suppressant treatment on the Th17/Treg balance in patients with CTD-aPAH.

## Figures and Tables

**Table 1:** Baseline clinical characteristics of study cohort.

Variable	CTD (*n* = 117)	CTD-aPAH			*P* value′	*P* value′′
		Total (*n* = 53)	Mild to moderate (*n* = 22)	Severe (*n* = 31)		
Gender						
Female (%)	81.2% (95)	79.2% (42)	77.3% (17)	80.6% (25)	0.766	0.765
Age (years)	61.0 ± 9.6	63.9 ± 8.5	64.0 ± 9.9	63.8 ± 7.5	0.058	0.921
BMI (kg/m^2^)	26.8 ± 2.6	26.1 ± 2.9	26.0 ± 3.2	26.2 ± 2.6	0.155	0.738
FEV1%	78.5 ± 10.4	71.3 ± 8.7	74.1 ± 6.3	69.2 ± 9.7	<0.01	0.030
DL_CO_%	72.2 ± 8.5	68.3 ± 7.8	71.0 ± 8.4	66.5 ± 6.9	<0.01	0.037
mPAP (mmHg)	21.0 ± 4.0	52.0 ± 8.5	48.9 ± 9.9	54.3 ± 6.6	<0.01	0.032
sPAP (mmHg)	33.7 ± 5.0	71.0 ± 9.2	67.7 ± 7.0	73.4 ± 7.7	<0.01	0.009
PCWP (mmHg)	—	12.2 ± 1.3	12.0 ± 1.3	12.3 ± 1.3	—	0.385
CI (L/min/m^2^)	2.9 ± 3.2	2.6 ± 3.3	2.8 ± 0.3	2.5 ± 0.3	<0.01	0.005
6MWD (m)	380.3 ± 75.1	294.3 ± 40.7	307.8 ± 42.0	284.7 ± 37.4	<0.01	0.041
WHO functional class						
I (%)	76.1% (89)	35.8% (19)	54.5% (12)	22.6% (7)	<0.01	0.017
II-III (%)	23.9% (28)	64.2% (34)	45.5% (10)	77.4% (24)	<0.01	0.017
Raynaud's phenomenon (%)	41.0% (48)	58.5% (31)	40.9% (9)	71.0% (22)	0.034	0.029
Cause of CTD						
SS	29	31	12	19		
SLE	42	6	2	4		
MCTD	22	13	4	9		
RA	21	1	0	1		
pSS	3	2	1	1		

*P* value′**: **CTD versus total CTD-aPAH; *P* value′′**: **mild to moderate CTD-aPAH versus severe CTD-aPAH. Data were presented as mean ± SD, *n* (%).

CTD: connective tissue diseases; CTD-aPAH: connective tissue diseases-associated pulmonary arterial hypertension; BMI: body mass index; FEV1%: forced expiratory lung volume in 1s (% predicted); DL_CO_%: carbon monoxide diffusion capacity (% predicted); mPAP: mean pulmonary artery pressure; sPAP: systolic pulmonary artery pressure; PCWP: pulmonary capillary wedge pressure; CI: cardiac index; 6MWD: 6-minute walk test distance; SS: systemic sclerosis; SLE: systemic lupus erythematosus; MCTD: mixed connective tissue disease; RA: rheumatoid arthritis; pSS: primary Sjogren's syndrome.

**Table 2:** Concentrations of serum cytokines and NT-proBNP determined by radioimmunoassay or Elecsys NT-proBNP sandwich immunoassay (pg/mL).

Variable	Control (*n* = 48)	CTD (*n* = 117)	CTD-aPAH		
			Total (*n* = 53)	Mild to moderate (*n* = 22)	Severe (*n* = 31)
IL-17a	56.54 ± 13.95	65.47 ± 19.77∗	74.68 ± 25.28^∗#^	66.50 ± 21.40	80.48 ± 26.52^§^
IL-6	44.48 ± 15.45	86.05 ± 35.83∗	97.26 ± 39.34∗	88.00 ± 38.40	103.84 ± 39.28
IFN-*γ*	329.52 ± 72.73	1002.62 ± 273.61∗	1083.08 ± 275.64∗	1014.59 ± 283.02	1131.68 ± 264.09
IL-2	349.73 ± 82.13	686.48 ± 183.92∗	719.53 ± 241.91∗	695.91 ± 231.51	736.29 ± 251.43
TNF-*α*	98.13 ± 25.42	329.65 ± 102.05∗	361.34 ± 118.41∗	318.05 ± 118.44	352.55 ± 121.98
TGF-*β*	126.29 ± 27.12	93.91 ± 21.50∗	71.17 ± 18.95^∗#^	77.50 ± 18.65	66.68 ± 18.13^§^
NT-pro BNP	103.67 ± 41.96	215.94 ± 78.20∗	453.04 ± 149.50^∗#^	402.91 ± 145.93	488.61 ± 143.81^§^

∗Compared with control group, *P* < 0.01; ^#^compared with CTD group, *P* < 0.01; ^§^compared with mild to moderate CTD-aPAH group, *P* < 0.01.
